# The role of sustainability integration into the corporate strategy – A perspective on analysts’ perceptions and buy recommendations

**DOI:** 10.1016/j.heliyon.2024.e25008

**Published:** 2024-01-20

**Authors:** Toni W. Thun, Anne Schneider, Christoph Kayser, Henning Zülch

**Affiliations:** Chair of Accounting and Auditing, HHL – Leipzig Graduate School of Management, Jahnallee 59, 04109, Leipzig, Germany

**Keywords:** Sustainability strategy, Sustainability integration, Corporate strategy, Analysts' perceptions, Analysts' recommendations

## Abstract

Sustainability is playing an increasingly important role in analysts' assessments of companies. Companies can address this importance through a sustainability strategy by choosing between a sustainability strategy independent from the corporate strategy (standalone sustainability strategy) or integrating sustainability into their corporate strategy (integrated strategy). For this purpose, we investigate the effects of different stages of sustainability integration into the corporate strategy on analysts' perceptions and buy recommendations. Our results show that analysts favour an integrated strategy over a standalone sustainability strategy. This finding is reflected in higher analysts’ perceptions of an integrated strategy. While a standalone sustainability strategy leads to fewer buy recommendations, an integrated strategy does not affect buy recommendations. We discuss our findings in relation to stakeholder theory and voluntary disclosure theory. Our study contributes to understanding how analysts perceive different stages of integrating sustainability into the corporate strategy. This understanding is relevant as analysts work as intermediaries in the capital market between companies and investors.

## 1 Introduction

For some years, sustainability aspects' relevance has grown in socio-political, economic and academic contexts. As a result, sustainability has become a competitive criterion for companies in the capital market due to rising stakeholder demands [[1], [2], [3]]. Companies' sustainability initiatives are essential in this connection as they lead to better financial access [4]. Financial market participants, including investors and analysts, pay greater attention to sustainability. The tendency is reflected in investors’ investment decisions gearing increasingly towards sustainability. Sustainability criteria are a priority for institutional investors, as evidenced by the fact that 75 % already use a sustainability integration strategy for their investment decisions [5].

The trend towards sustainable investment is growing in Europe, driven by political incentives of the European Union, including the Action Plan on Sustainable Finance, which leads to an increase in analysts' and investors’ requirements for sustainability information [6]. Within financial markets, analysts act as intermediaries of information [7], linking financial and sustainability performance by processing relevant sustainability information [8]. This intermediary role is important for the capital markets participants. However, a need remains to understand how analysts evaluate sustainability information [7].

Analysts use sustainability information to assess a company's sustainability performance, which is why companies need to provide sustainability information for more accurate analyst evaluations [9]. Moreover, disclosing sustainability information positively impacts analyst coverage [[10], [11], [12]] and recommendations [8,13]. Furthermore, analysts increasingly emphasise the value-enhancing role of sustainability information in their company valuations [[14], [15], [16], [17]]. Additionally, research indicates that the improved provision of sustainability information leads to more precise analyst earnings forecasts [15,18].

According to stakeholder theory, companies should provide their stakeholders with the necessary sustainability information [[19], [20], [21]]. Prospective information is important for analysts' assessments [22], which can be provided by disclosing a company's strategy. An appropriate strategy is fundamental to implementing sustainability activities [23]. Voluntary disclosure of combined financial and non-financial information has positive effects on investors' perceptions [24], improves analysts' forecasts [25] and decreases companies' costs of debt [26].

Ultimately, companies have to choose how to integrate sustainability into their corporate strategy. They can either adopt a sustainability strategy independent of their corporate strategy (standalone sustainability strategy) or integrate sustainability into their corporate strategy (integrated strategy). Capital market participants highly desire integrated sustainability information [19]. Eventually, integrating sustainability into strategic corporate governance expresses the company's ability to deal with sustainability [19]. Additionally, implementing a sustainability strategy positively affects sustainability performance [[27], [28], [29], [30], [31], [32]]. Moreover, there is evidence that sustainability strategies positively influence financial performance [[33], [34], [35], [36], [37], [38], [39]]. In addition, a corporate strategy has a moderating effect on sustainability and the company's performance [40].

Accordingly, institutional theory can rationalise why analysts respond to certain disclosure practices and why companies implement certain activities or practices [41]. Analysts can exert pressure on companies, especially as financial market intermediaries, e.g., in adopting strategies [42]. Therefore, a standalone sustainability strategy or an integrated strategy can provide analysts with a direction for their perception and recommendation as a particular response. Therefore, the strategic orientation of a company plays an essential role in sustainable corporate management and creating sustainable value [43].

Although sustainability strategies are gaining momentum in academic research, most studies regarding analysts only consider sustainability information in general and do not focus on the impact of a sustainability strategy. Nevertheless, research suggests how companies should integrate sustainability aspects into their corporate strategies and sustainability strategies [43,44]. Furthermore, literature suggests that disclosing a sustainability strategy positively impacts analysts' assessments [7]. However, the impact of a standalone sustainability strategy or an integrated strategy on analysts remains unclear. This question is important for companies to understand as the different stages might impact analysts and their access to financial resources. As former studies focus on showing the influence of overall sustainability strategy on performance [1,21,31,34,40,45] or the impact of corporate strategies on analysts' assessment [[46], [47], [48]], we investigate the relationship of the sustainability integration into a strategy on analysts' perceptions and buy recommendations. We aim to examine different stages of integrating sustainability into the strategies. For this purpose, we are one of the first studies to investigate the influence of two approaches to consider sustainability strategically, namely a standalone sustainability strategy and an integrated strategy, on analysts' perceptions and buy recommendations. Especially against the background that only a general context is shown in the literature, our research provides a concrete insight into whether the different stages are meaningful for analysts by measuring analysts’ perceptions using a unique data set. Accordingly, we investigate the following research question.

(RQ) How do analysts perceive different stages of integration of sustainability into the corporate strategy?

To answer our RQ, we categorise the integration of sustainability into corporate strategy into two phases: a standalone sustainability strategy and an integrated strategy. In the first stage, sustainability is not part of the corporate strategy. However, a standalone sustainability strategy exists independent of the corporate strategy and is mostly presented in the sustainability report. In the second stage, sustainability is integrated into the corporate strategy. We use the sustainability and annual reports of publicly listed German companies from 2017 to 2021 to determine the stages of their sustainability strategies. As part of the annual report, the German management report provides a regulatory setting specifying elements if voluntary strategy reporting is chosen that enables us to identify these strategies. Furthermore, we conducted surveys with analysts operating in the German capital market, gaining qualitative data on the perception of the sustainability strategy. Finally, regression analysis is applied to test the impact of the two stages of sustainability integration on analysts’ perceptions and buy recommendations.

Our findings demonstrate that analysts prefer an integrated strategy over a standalone sustainability strategy. Furthermore, analysts show a reluctance to a standalone sustainability strategy by having a lower perception of the strategy itself and giving fewer buy recommendations. In addition, even though analysts acknowledge an integrated strategy, this acknowledgement does not translate into more buy recommendations. Nevertheless, the integration promotes analysts' confidence in a company's responsible approach to sustainability.

We contribute to the existing literature by evidencing how analysts perceive sustainability information by focusing on different stages of sustainability integration into the corporate strategy, as no studies deal with analysts and their opinions on sustainability strategies. Accordingly, we focus on sustainability strategy as part of the sustainability information environment and, therefore, prospective information issued by the companies. Second, we provide companies with insights into the impact different stages of integrating sustainability into the corporate strategy have on analysts’ opinions. In addition, we provide other capital market participants, such as investors, with further knowledge about how sustainability and strategy are perceived and evaluated by analysts.

The paper is structured as follows: After this introduction, section 2 derives the hypothesis based on existing literature and theory. Section 3 comprises the methodology and the data sample. In the fourth section, the results are presented, and section 5 discusses the findings. The paper ends with a conclusion, including further research and implications.

## 2 Literature review and hypotheses development

In the light of stakeholder theory, a company's management must consider the interests of every target group [49,50], especially in disclosing sustainability information [51,52]. Precisely, analysts emphasise the quality of sustainability information because they can draw meaningful information from them [8,41,53,54]. Therefore, analysts demand sustainability information from companies, especially in using sustainability reports [15]. According to stakeholder theory, sustainability in a company and its voluntary disclosure of sustainability information can enhance reputation, which can improve financial performance, lead to more accurate earnings forecasts and analyst recommendations [55].

Strategic planning on sustainability issues enables companies to address sustainability aspects and develop effective responses proactively [56]. Consequently, strategic planning enhances social responsibility and competitive advantage by raising awareness of stakeholder needs [44]. As analysts need to take a long-term and forward-looking view, a sustainability strategy illustrates that companies have targeted and future-oriented plans regarding sustainability practices [57]. Consequently, analysts value strategic orientations regarding sustainability, as a clear strategy provided by companies facilitates their understanding and evaluation of information [58].

The impact of sustainability strategies has been studied in many ways. In general, a sustainability strategy can help companies to integrate sustainability into their management, i.e., into actions, business practices and decision-making [59]. Initially, a sustainability strategy positively impacts a company's financial performance [1,34,38,39,60] and the implementation of sustainability innovations leading to improved financial performance [36]. A sustainability strategy shows analysts that companies can manage external threats [57,61,62] and are more resilient in their long-term financial performance [63]. Cheng et al. (2014) [13] argue that implementing sustainability strategies enables companies to improve their access to financial resources. Additionally, companies with a sustainability strategy demonstrate better sustainability performance [32,[64], [65], [66]]. In this way, companies can convince analysts of their sustainability strategy, whereby analysts primarily rely on qualitative and transparent information when evaluating strategies [67].

Considering stakeholder theory, it is crucial to satisfy the needs of stakeholders through sustainability disclosure [7]. Analysts are getting better at assessing sustainability ratings by understanding how sustainability can contribute to value creation in a company [13]. Accordingly, analysts increasingly emphasise the value-enhancing role of sustainability information, which includes information on the sustainability strategy, in their company valuations [[14], [15], [16], [17],^68^]. By disclosing a sustainability strategy, a company could fulfil the expectations and demands of analysts, which can result in more beneficial recommendations [7].

Drawing on stakeholder theory and recognizing the significance of a strategic orientation towards sustainability, we derive the following hypotheses regarding the impact on analysts when an independent sustainability strategy (standalone sustainability strategy) exists.

H1a A standalone sustainability strategy is positively related to analysts' perceptions.

H1b A standalone sustainability strategy is positively related to analysts' buy recommendations.

One option to anchor sustainability in the corporate organisation is a strategy combining sustainability aspects with business matters [19]. For this purpose, sustainability should be seen as a holistic approach within a company, including strategic decisions that consider stakeholder concerns [23]. A holistic view is important, as a company's strategic orientation shows analysts that companies are addressing both environmental and social aspects and thus integrating sustainability into their strategic decisions [69]. Consequently, sustainability integration can reflect positively on analysts' perceptions. For example, analysts attach importance to sustainability measures being incorporated into strategic developments and responsibility for sustainability being clearly defined [69]. Further, there is evidence that integrating sustainability in the company's management leads to better sustainability performance [70] and sustainability as part of the company's strategy leads to better financial performance [37,71]. Furthermore, an integrated view of sustainability increases stock returns and strengthens the relationship with stakeholders [54].

While recent literature finds that disclosure of sustainability strategy information is important to analysts [11,13,15], additional evidence is that how disclosure is provided adds even more value for analysts. One step is to offer analysts information on sustainability integrated into the corporate strategy (integrated strategy). By integrating sustainability into the corporate strategy and stakeholder management, companies can better address the interests of stakeholders, including analysts [62]. Disclosing combined financial and sustainability information is a coherent approach that promotes the effectiveness of sustainability information for the benefit of stakeholders [72]. In general, integrated disclosure has a positive impact on a company's reputation and financial performance [73], company value [72,74], and profitability [26]. Furthermore, integrated disclosure creates more transparency, as information asymmetries can be reduced [24,75,76]. As integrated disclosure is often associated with voluntary disclosure theory, it can be argued that stakeholders, such as analysts, highly appreciate when companies voluntarily integrate sustainability information into corporate strategy [7,77].

Accordingly, the integrated presentation of sustainability information in a strategic context is perceived positively by analysts as it can reduce information asymmetries [78] and provide helpful information for the capital market [74,79]. In particular, the combined provision of financial and sustainability information enables analysts to make more accurate forecasts [25,77,80]. Moreover, the strategic provision of sustainability information leads to more favourable market reactions from analysts [81]. When companies integrate sustainability into their corporate strategy, they can show responsibility and convey an authentic corporate image [56,62].

So, companies should be interested in convincing analysts that sustainability actions result in future profitability in the long run [54], as analysts are interested in companies' future sustainable development. As analysts serve as intermediaries on the capital market [7], companies should dissuade them from giving unfavourable recommendations because of their impact on investors’ decision-making [54]. In addition, companies can provide analysts with valuable information by offering sustainability strategies, as García-Sánchez et al. (2020) [41] demonstrated for strategies related to Sustainability Development Goals.

Since we assume that an integrated strategy is a unique strategy, we argue that companies can use it to profile themselves on the capital market to analysts [47]. By combining the increasing demands of sustainability information from analysts [6,14,82] and the importance of corporate strategies, including integrating sustainability aspects [47,48,56,61,83], we argue that integrating sustainability into the corporate strategy shows a genuine commitment to sustainability recognised by analysts. Accordingly, we formulate the following hypotheses.

H2a The integration of sustainability into the corporate strategy is positively related to analysts' perceptions.

H2b The integration of sustainability into the corporate strategy is positively related to analysts' buy recommendations.

## 3 Research design

### 3.1 Sample selection

The sample comprises publicly listed German companies in the DAX, MDAX and SDAX between 2017 and 2021. This period ensures a consistent regulatory environment for disclosing sustainability information regarding the sustainability report in Germany since the European Non-Financial Reporting Directive (NFRD) 2014/95/EU came into effect in 2017. Regarding the sample, we argue that Germany is a suitable example, as the NFRD proposes strategies for presenting sustainability aspects. Additionally, the German Accounting Standard 20 (GAS 20) also provides guidelines on disclosing the corporate strategy within the group's management report. Both regulations allow us to identify companies' sustainability strategies by analysing sustainability and annual reports.

Our analysis depends on the qualitative perceptions of analysts on a company's sustainability strategy. For this purpose, a survey was conducted annually from mid-April to mid-June after the annual reports for the previous year had been published, asking analysts to assess the companies' sustainability strategy. Accordingly, we obtain analysts' perceptions of the sustainability strategy of the previous year, as annual reports were published by the end of April. We set the minimum number of responses by analysts per company and year to three so that this company-year observation is considered in the analysis. The initial sample thus amounts to 694 observations.

We then use annual and sustainability reports to collect information on sustainability strategies. Missing data in the Refinitiv Database, which is used to obtain financial data, including information on buy recommendations, leads to a final sample of 600 observations.

### 3.2 Variables

#### 3.2.1 Dependent variables

Our first dependent variable is analysts' perceptions of the sustainability strategy. As mentioned above, analysts are asked to assess the companies' sustainability strategy yearly. In order to obtain a broad and representative coverage of the analysts' assessment, banks, asset managers and other institutions with businesses in Germany that provide analysis on the companies reported are contacted. Each year, the analysts receive a questionnaire^1^ in which they rate the sustainability strategy of each company listed in the DAX, MDAX or SDAX on a scale of 1–6, where 1 stands for ‘very positive’ and 6 for ‘very negative’. The question is formulated as follows: *How do you evaluate the company's sustainability strategy?*

We apply a single-item measure in our research to mitigate the risk of inadequate response effort behaviours, such as straight-lining or non-response errors. Furthermore, it guarantees that we receive input on a vast array of companies and, thus, increase our evaluated sample size within our sample of 160 firms. Research in business and psychology research support the usability and predictive validity of single-item measures [84,85].

Also, we do not distinguish between buy- and sell-side analysts, as no differences in their assessments are expected regarding the sustainability strategy [86]. Not every analyst contacted provides an evaluation for every company; therefore, only those companies-year observations for which at least three analysts' evaluations per year are available are considered. The analysis uses the mean value of the analysts’ evaluations. The evaluation is then converted from an ordinal scale to an interval scale (0–100) via a minimum and maximum normalisation (rescaling) of the yearly average rating by following formula:

(1) $$Normalised\;Value=-\;\frac{x-\min\left[x\right]}{span\left(\max\left[x\right],\min\left[x\right]\right)},with\;\max\left[x\right]=1,\min\left[x\right]=6$$

To get the variable *StratPerception*, we use the following derived formula for company *i* and year *t*:

(2) $${\text{StratPerception}}_{\text{it}}=\left(1.2-0.2*{\text{Mean}\;\text{of}\;\text{analyst}s^{\prime }\text{perception}}_{\text{it}}\right)*100$$

The score has a scale from 0 to 100. An average rating of 1 (‘very positive') is assigned a score of 100 and an average rating of 6 (‘very negative') is assigned a score of 0. The conversion is done on a linear basis. The higher the score, the better the sustainability strategy is perceived by analysts. Accordingly, *StratPerception* represents a qualitative assessment of the sustainability strategy by analysts.

The second dependent variable is the buy recommendations provided by analysts. *Buy* is measured as the percentage of buy recommendations to the total recommendations one month after the publication of the annual report. Therefore, *Buy* scales from 0 to 100, where 0 means there are no buy recommendations and 100 means all recommendations are buy recommendations. The investment opinion of analyst recommendations is a widely used method in financial analyst research [87]. Table 1 shows all variables used and their definitions.

**Table 1:** Variables overview.

Variable	Description	Source
Dependent Variables		
StratPerception	Score of analyst's perception of sustainability strategy	Hand collected
Buy	Percentage of Buy Recommendations	Refinitv
Independent Variables		
StandaloneStrat	Dummy variable, which takes the value 1 if the company has a separate sustainability strategy and 0 otherwise	Hand collected
IntegratedStrat	Dummy variable, which takes the value 1 if sustainability is integrated into the corporate strategy and 0 otherwise	Hand collected
Control Variables		
Size	The natural logarithm of market capitalisation	Refinitv
RoA	Return on Assets	Refinitv
Leverage	Ratio of total debt to total capital	Refinitv
BTM	Book to market value	Refinitv
FCF	Free Cash Flow	Refinitv
Blockholders	The percentage of shares held by management and outside shareholder with at least 5 % ownership	Refinitv
BoardInd	Percentage of independent board members as reported by the company	Refinitv
ESG-Score	Refinitiv's ESG overall company score	Refinitv

#### 3.2.2 Independent variables

Our variables of interests are the stages of integration of sustainability into the corporate strategy and buy recommendations. For the analysis, we distinguish between two stages of integration. In the first stage, sustainability is not part of the corporate strategy. However, there is an independent sustainability strategy (standalone sustainability strategy). In the second stage, sustainability is integrated as an aspect into the corporate strategy (integrated strategy).

Information on the integration of sustainability into the corporate strategy is hand-collected from the annual and sustainability reports of German companies listed in DAX, MDAX and SDAX from 2017 to 2021. For this purpose, the management report as part of the annual report is checked for a section on corporate strategy. If a company decides to disclose its strategy, the German Accounting Standard 20 (GAS 20) provides guidelines on what information companies have to disclose, such as stating the scope and timing of the strategic objectives.

Accordingly, sustainability is deemed integrated into the corporate strategy if it is included in the annual report's corporate strategy section and presented as an integral part of the corporate strategy. We define sustainability aspects as an integral part of the corporate strategy if they are presented following the guidelines of GAS 20. We define the variable *IntegratedStrat* as 1 if their sustainability is integrated into the corporate strategy and 0 otherwise.

If there is no section on corporate strategy in the annual report or sustainability is not included in the corporate strategy, the sustainability report is examined to determine if a sustainability strategy exists. Suppose a sustainability strategy is disclosed in the sustainability report, and the disclosure follows the guidelines of GAS 20. In that case, it is considered a standalone sustainability strategy. The variable *StandaloneStrat* takes the value 1 if there is a standalone sustainability strategy and 0 otherwise.

#### 3.2.3 Control variables

To complement the analysis, we use several control variables throughout our regression models that might affect analysts' perceptions of sustainability strategy and their recommendations to prevent estimation bias. First, we start with companies' financial characteristics. Ioannou and Serafeim (2015) [54] argue that analysts might favour larger firms. Accordingly, we control for the firm's size (*Size*) using the natural logarithm of market capitalisation [41,55]. To control for profitability, like in most studies, return on assets (*RoA*) is added [8,53]. *Leverage* is measured as the ratio of total debt to total capital [41,55]. In addition to the natural logarithm of market capitalisation, we add the ratio of book to market value (*BTM*) [54,88].

Furthermore, we use free cash flow (*FCF*) as an indicator of free funds to pursue the sustainability strategy, which might come with additional costs and investments [89]. The proportion of shares owned by management and outside shareholders who possess at least 5 % of the company (*Blockholders*) is added as an ownership variable. We argue that blockholders have been involved in the strategy development process and are, therefore, more likely to support the management in implementing the communicated strategy. Consequently, analysts should have more confidence in the sustainability strategy with a larger share of blockholders. The findings of Luo et al. (2015), Wan-Hussin et al. (2021) and Gu et al. (2013) [8,55,88] support this argumentation. García-Sánchez et al. (2020) [41] show a positive impact of board independence on analyst recommendations. Accordingly, we add *BoardInd*, measured as the percentage of independent board members.

Finally, we further control for the sustainability performance as an indicator of the past execution of the sustainability strategy [30,32] and add the environmental, social and governance score from the Refinitiv Database (*ESG-Score*).

### 3.3 Regression models

We use fixed-effect panel regression models to test our hypotheses. The regression model to analyse the effect on analysts’ perceptions of the sustainability strategy for company *i* and year *t* is:

(3) $${\text{StratPerception}}_{\text{it}}=\alpha_{0}+\beta_{1}{\text{SustainabilityStrategy}}_{\text{it}}+\beta_{2}{\text{Size}}_{\text{it}}+\beta_{3}{\text{RoA}}_{\text{it}}+\beta_{4}{\text{Leverage}}_{\text{it}}+\beta_{5}{\text{BTM}}_{\text{it}}+\beta_{6}{\text{FCF}}_{\text{it}}+\beta_{7}{\text{Blockholders}}_{\text{it}}+\beta_{8}{\text{BoardInd}}_{\text{it}}+\beta_{9}{\text{ESG}-\text{Score}}_{\text{it}}+\text{Year}\;\text{dummies}+\varepsilon_{\text{it}}$$

For the percentage of buy recommendations, the following regression model is constructed:

(4) $${\text{Buy}}_{\text{it}}=\alpha_{0}+\beta_{1}{\text{SustainabilityStrategy}}_{\text{it}}+\beta_{2}{\text{Size}}_{\text{it}}+\beta_{3}{\text{RoA}}_{\text{it}}+\beta_{4}{\text{Leverage}}_{\text{it}}+\beta_{5}{\text{BTM}}_{\text{it}}+\beta_{6}{\text{FCF}}_{\text{it}}+\beta_{7}{\text{Blockholders}}_{\text{it}}+\beta_{8}{\text{BoardInd}}_{\text{it}}+\beta_{9}{\text{ESG}-\text{Score}}_{\text{it}}+\text{Year}\;\text{dummies}+\varepsilon_{\text{it}}$$

Both regression models are run with year-fixed effects. For *SustainabilityStrategy*, the two variables of the integration of sustainability strategy (*StandaloneStrat* and *IntegratedStrat*) are used.

## 4 Empirical results

### 4.1 Descriptive and correlation statistics

Descriptive statistics for all variables are shown in Table 2. The range of the sustainability strategy perceptions ranges from 26.67 to 95. The mean of 65.19 shows that analysts are only partially satisfied with the companies’ sustainability strategies. The mean of 50.58% of buy recommendations indicates that even though analysts are not completely satisfied with the sustainability strategies, they might recommend buying shares of the companies. The results show that 51% of the observations disclose a standalone sustainability strategy in their sustainability report. In comparison, 45% of the observations provide information on sustainability as part of the corporate strategy.

**Table 2:** Descriptive statistics.

	N	Mean	S.D.	Min	Q1	Median	Q3	Max
Dependent Variables								
StratPerception	600	65.19	9.79	26.67	60.00	65.86	71.67	95.00
Buy	600	50.58	23.33	0.00	33.34	50.00	66.67	100.00
Independent Variables								
StandaloneStrat	600	0.51	0.50	0.00	0.00	1.00	1.00	1.00
IntegratedStrat	600	0.45	0.50	0.00	0.00	0.00	1.00	1.00
Control Variables								
Size	600	15.40	1.39	12.37	14.35	15.27	16.35	18.81
RoA	600	4.88	7.01	−31.44	1.92	4.58	7.72	80.13
Leverage	600	37.85	21.89	0.00	19.74	37.81	53.00	91.73
BTM	600	0.63	0.59	0.03	0.25	0.47	0.79	4.34
FCF	600	0.42	3.30	−10.77	−0.12	0.04	0.26	33.95
Blockholders	600	31.50	25.25	0.00	5.86	30.73	50.52	96.40
BoardInd	600	55.56	33.99	0.00	28.57	53.59	88.89	100.00
ESG-Score	600	61.25	19.37	9.09	47.76	63.26	76.93	94.81

Table 3 reports the correlation coefficients between all variables used. The correlation matrix does not provide evident signs of multicollinearity between the variables. Nevertheless, the independent variable (*IntegratedStrat*) and dependent variable (*StratPerception*) are significantly positively correlated. To ensure multicollinearity is not an issue in our analysis, we further compute the variance inflation factors (VIFs) for all regressions. The VIFs (all values below 3) do not suggest multicollinearity to be a major concern in our analysis.

**Table 3:** Correlation table.

	StratPerception	Buy	Standalone-Strat	IntegratedStrat	Size	RoA	Leverage	BTM	FCF	Blockholders	BoardInd	ESG-Score	Strategy-Disclosure	MTBV	CapIn	FreeFloat	Big4
StratPerception	1	0.110*	−0.005	0.103*	0.202*	0.324*	−0.102*	−0.320*	0.157*	−0.021	0.107*	0.105*	0.070*	0.295*	0.135*	0.020	0.037
Buy	0.091*	1	0.046	0.005	0.004	0.071*	0.069*	−0.002	0.047	−0.215*	0.155*	0.015	−0.005	0.017	0.148*	0.129*	−0.166*
StandaloneStrat	0.002	0.042	1	0.065	0.040	−0.139*	0.038	0.045	−0.046	−0.009	0.061	0.140*	0.083*	−0.061	−0.026	−0.048	0.086*
IntegratedStrat	0.122*	0.003	0.065	1	0.337*	−0.007	0.086*	0.007	0.089*	−0.034	0.000	0.430*	0.383*	−0.005	−0.029	0.079*	0.136*
Size	0.197*	0.017	0.029	0.339*	1	0.082*	0.102*	−0.140*	0.285*	−0.050	0.199*	0.628*	0.112*	0.118*	0.095*	0.110*	0.317*
RoA	0.247*	0.029	−0.093*	0.002	0.069*	1	−0.285*	−0.367*	0.166*	0.005	0.102*	−0.102*	0.162*	0.396*	0.148*	0.005	−0.074*
Leverage	−0.109*	0.053	0.033	0.075*	0.102*	−0.249*	1	0.124*	−0.032	−0.153*	0.060	0.202*	0.078*	−0.160*	−0.032	0.137*	0.085*
BTM	−0.303*	−0.094*	−0.028	−0.019	−0.096*	−0.169*	0.236*	1	−0.163*	−0.111*	−0.081*	0.074*	−0.130*	−0.962*	−0.331*	0.155*	−0.021
FCF	0.096*	0.097*	−0.058	0.122*	0.278*	−0.021	−0.033	−0.008	1	0.003	0.011	0.129*	−0.003	0.154*	0.190*	0.015	0.035
Blockholders	−0.022	−0.218*	−0.007	−0.022	−0.067*	0.005	−0.154*	−0.097*	−0.063	1	−0.300*	−0.258*	0.001	0.121*	0.012	−0.826*	−0.071*
BoardInd	0.086*	0.134*	0.064	0.003	0.225*	0.058	0.059	−0.053	−0.041	−0.299*	1	0.245*	0.164*	0.078*	0.117*	0.248*	0.063
ESG-Score	0.084*	−0.013	0.160*	0.430*	0.618*	−0.074*	0.188*	0.046	0.192*	−0.239*	0.260*	1	0.161*	−0.096*	0.021	0.295*	0.306*
StrategyDisclosure	0.077*	−0.009	0.083*	0.383*	0.100*	0.139*	0.065	−0.129*	0.013	0.010	0.168*	0.208*	1	0.116*	0.159*	0.042	0.101*
MTBV	0.229*	0.009	−0.109*	−0.084*	0.066	0.233*	−0.058	−0.489*	−0.043	0.158*	0.107*	−0.078*	0.048	1	0.324*	−0.158*	0.016
CapIn	0.109*	0.134*	−0.039	−0.089*	0.121*	0.081*	−0.024	−0.310*	−0.025	0.017	0.141*	−0.019	0.151*	0.237*	1	−0.035	−0.087*
FreeFloat	0.012	0.143*	−0.056	0.047	0.115*	−0.001	0.136*	0.144*	0.105*	−0.853*	0.262*	0.267*	0.021	−0.125*	−0.019	1	0.084*
Big4	0.036	−0.184*	0.086*	0.136*	0.301*	−0.039	0.085*	−0.003	0.038	−0.058	0.072*	0.319*	0.101*	−0.029	−0.102*	0.045	1

Notes: Pearson correlation coefficients are shown below and Spearman correlations coefficients above the diagonal. * indicates a significance level of 10 %.

### 4.2 Empirical analysis of stages of sustainability integration

Table 4 presents the results of the different stages of sustainability integration into the corporate strategy on the analysts' perceptions of the sustainability strategy and the percentage of buy recommendations. Contrary to our expectation and Hypothesis 1a, a standalone sustainability strategy does not lead to a higher sustainability strategy perception (model (1)). With a coefficient of −1.950 (p < 0.05), a standalone sustainability strategy is even negatively related to analysts’ perceptions. This result does not change if sustainability integrated into the corporate strategy is considered in the regression model (model (3): β = −1.921, p < 0.05).

**Table 4:** Empirical results on sustainability strategy on analysts.

Dependent Variable	StratPerception			Buy		
Model	(1)	(2)	(3)	(4)	(5)	(6)
StandaloneStrat	−1.950**		−1.921**	−7.871***		−7.850***
	(0.035)		(0.036)	(0.000)		(0.000)
IntegratedStrat		2.931***	2.904***		2.283	2.171
		(0.008)	(0.009)		(0.383)	(0.400)
Size	1.788	1.627	1.730	−0.302	−0.763	−0.345
	(0.168)	(0.207)	(0.179)	(0.920)	(0.803)	(0.909)
RoA	0.131**	0.127**	0.125**	0.155	0.157	0.15
	(0.023)	(0.028)	(0.029)	(0.248)	(0.248)	(0.263)
Leverage	0.027	0.037	0.03	−0.108	−0.075	−0.106
	(0.479)	(0.320)	(0.427)	(0.218)	(0.397)	(0.229)
BTM	−0.412	−0.449	−0.431	−2.765	−2.853	−2.779
	(0.789)	(0.771)	(0.779)	(0.442)	(0.434)	(0.439)
FCF	0.683***	0.692***	0.658***	0.022	0.145	0.003
	(0.001)	(0.001)	(0.001)	(0.962)	(0.762)	(0.994)
Blockholders	0.142***	0.141***	0.138***	−0.251**	−0.241**	−0.254**
	(0.001)	(0.001)	(0.002)	(0.014)	(0.021)	(0.013)
BoardInd	−0.003	0.006	0.006	0.003	0.010	0.010
	(0.925)	(0.823)	(0.819)	(0.958)	(0.885)	(0.877)
ESG-Score	−0.046	−0.049	−0.045	−0.101	−0.119	−0.100
	(0.399)	(0.364)	(0.410)	(0.428)	(0.357)	(0.434)
Intercept	39.26*	39.471*	38.781*	74.802	77.263	74.443
Year Fixed Effects	Yes	Yes	Yes	Yes	Yes	Yes
N	600	600	600	600	600	600
r^2^	0.163	0.168	0.177	0.158	0.133	0.160
F-Statistic	6.336***	6.569***	6.464***	6.098***	4.971***	5.709***

Notes: p-values in parentheses. ***, ** and * indicates a significance level of 1 %, 5 % and 10 %, respectively.

However, the integration of sustainability into the corporate strategy has a positive effect on analysts’ perceptions. The positive impact can be observed when *IntegratedStrat* is added as a single variable (model (2): β = 2.931, p < 0.01) and when simultaneously added with *StandaloneStrat* (model (3): β = 2.904, p < 0.01). Therefore, the results support Hypothesis 2a. The aversion to a standalone strategy may be due to a need for more credibility and relevance of a sustainability strategy if it is separate from the corporate strategy.

While there is a clear relationship between the integration status of sustainability strategy and analysts' perceptions, this is not the case for buy recommendations, as Table 4 shows (model (4) to (6)). In line with the results of analysts' perceptions and in contrast with our hypothesis H1b, a standalone sustainability strategy does not increase but decreases the percentage of buy recommendations. The coefficients of −7.871 (model (4); p < 0.01) and −7.850 (model (6); p < 0.01) suggest a strong aversion of analysts to a standalone sustainability strategy. Furthermore, the regression results show no significant relationship between an integrated strategy and buy recommendations. Thus, we reject hypothesis H2b. An integrated strategy might add to the corporate strategy's uniqueness and enrich the information environment, creating information costs for analysts [48], making it challenging to consider it in their company valuations.

Comparing our r-squared with other studies dealing with analyst perceptions and recommendations, we conclude that the r-squared is the same size as other studies [54,55,88].

### 4.3 Robustness tests

#### 4.3.1 Addressing self-selection bias: heckman correction

The analysis might be subject to a selection bias due to the voluntary nature of strategy disclosure in Germany. Companies might be reluctant to disclose their strategy even though investors want to know it [[90], [91], [92]]. Though most companies should have a corporate strategy, some do not disclose their strategy to prevent competitors from copying relevant strategic key elements or anticipating their strategic action plan [91,93]. Additionally, management might choose to disclose their strategy as a signalling effect to differentiate themselves from competitors and other companies [94]. As a result, management has several reasons for disclosing or not disclosing the company's strategy.

Relying on sustainability and corporate strategy disclosure, the analysis faces possible self-selection bias. To control for this issue, we employ the two-stage Heckman correction [95]. The first stage models that a company discloses its corporate strategy. Based on the findings of Winter and Zülch (2019) [90] for the German capital market and its determinants of voluntary strategy disclosure, *MTBV* (market to book value), *CapIn* (ratio of intangibles to total assets), and *FreeFloat* (free float numbers of shares) are added as new variables supplementing *Size*, *RoA* and *Leverage*. Following Morris and Tronnes (2018) [91], *Big4* (a dummy variable that takes the value 1 if a Big 4 firm audited the annual report) is also used in the first stage regression. Considering these variables, the resulting probit model is formulated as follows:

(5) $${\text{StrategyDisclosure}}_{\text{it}}=\alpha_{0}+\beta_{1}{\text{Size}}_{\text{it}}+\beta_{2}{\text{RoA}}_{\text{it}}+\beta_{3}{\text{Leverage}}_{\text{it}}+\beta_{4}{\text{MTBV}}_{\text{it}}+\beta_{5}{\text{CapIn}}_{\text{it}}+\beta_{6}{FreeFloat}_{\text{it}}+\beta_{7}{\text{BoardInd}}_{\text{it}}+\beta_{8}{\text{Big}4}_{\text{it}}+\text{Year}\;\text{dummies}+\varepsilon_{\text{it}}$$

Based on the results of the first stage, the inverse-Mills ratio (*imr*) is calculated and then added as an additional variable to the regression models (1) and (2).

Table 5 presents the second stage's results for the analysts' perceptions and buy recommendations. The coefficients of *imr* are significant in the regressions with *StratPerception* as the dependent variable supporting our assumption that self-selection is present. The regression model with *Buy* as the dependent variable does not show a significant *imr*. Nevertheless, the results confirm the findings in section 4.2 regarding the variables of interest. A standalone strategy negatively impacts analysts' perceptions and buy recommendations. Additionally, an integrated strategy positively affects analysts' perceptions but does not affect buy recommendations. The coefficients are of a comparable order of magnitude to those in the main analysis.

**Table 5:** Heckman 2nd stage regression results.

Dependent Variable	StratPerception			Buy		
Model	(7)	(8)	(9)	(10)	(11)	(12)
StandaloneStrat	−1.825**		−1.802**	−7.740***		−7.724***
	(0.048)		(0.049)	(0.000)		(0.000)
IntegratedStrat		2.848**	2.828**		2.178	2.090
		(0.010)	(0.010)		(0.406)	(0.418)
Size	1.740	1.587	1.685	−0.352	−0.815	−0.392
	(0.178)	(0.217)	(0.189)	(0.907)	(0.790)	(0.896)
RoA	0.018	0.011	0.017	0.037	0.012	0.036
	(0.821)	(0.886)	(0.829)	(0.844)	(0.951)	(0.847)
Leverage	0.002	0.012	0.006	−0.134	−0.108	−0.131
	(0.952)	(0.762)	(0.869)	(0.148)	(0.251)	(0.158)
BTM	−0.319	−0.352	−0.341	−2.667	−2.730	−2.684
	(0.836)	(0.819)	(0.823)	(0.458)	(0.454)	(0.456)
FCF	0.67***	0.677***	0.645***	0.008	0.126	−0.010
	(0.001)	(0.001)	(0.002)	(0.986)	(0.793)	(0.984)
Blockholders	0.144***	0.143***	0.139***	−0.250**	−0.240**	−0.253**
	(0.001)	(0.001)	(0.001)	(0.015)	(0.021)	(0.014)
BoardInd	−0.026	−0.018	−0.016	−0.021	−0.021	−0.014
	(0.385)	(0.552)	(0.588)	(0.763)	(0.770)	(0.844)
ESG-Score	−0.042	−0.044	−0.040	−0.096	−0.112	−0.095
	(0.446)	(0.413)	(0.456)	(0.451)	(0.383)	(0.455)
imr	−10.036**	−10.235**	−9.582*	−10.483	−12.944	−10.148
	(0.044)	(0.039)	(0.053)	(0.366)	(0.271)	(0.382)
Intercept	45.03**	45.323**	44.303**	80.829*	84.665*	80.291*
Year Fixed Effects	Yes	Yes	Yes	Yes	Yes	Yes
N	600	600	600	600	600	600
r^2^	0.171	0.177	0.184	0.160	0.135	0.161
F-Statistic	6.218***	6.454***	6.325***	5.718***	4.705***	5.376***

Notes: p-values in parentheses. ***, ** and * indicates a significance level of 1 %, 5 % and 10 %, respectively.

#### 4.3.2 Instrumental variable regression

Furthermore, we address endogeneity concerns by performing two-stage least squared (2SLS) regression. We use the industry-year-mean of our independent variables as instrumental variables following prior research [71,96,97]. Given that our endogenous variable is binary, we follow the approach to run a probit model on the instrument and controls in the first step. Then use those predicted values in place of the instrument in the following 2SLS estimation [98]**.**

The results of the second stage instrumental variable regression are shown in Table 6. The results corroborate our previously shown findings. *StandaloneStrat* is negatively associated with the perception of the sustainability strategy and the number of buy recommendations of analysts. *IntegratedStrat* is positively associated with the perception of the sustainability strategy and does not show an influence on the number of buy recommendations.

**Table 6:** 2SLS regression results.

Dependent Variable	StratPerception			Buy		
Model	(13)	(14)	(15)	(16)	(17)	(18)
StandaloneStrat	−7.073*		−8.524*	−16.508*		−20.586**
	(0.089)		(0.055)	(0.083)		(0.038)
IntegratedStrat		9.632**	10.518**		16.145	13.079
		(0.013)	(0.012)		(0.121)	(0.160)
Size	2.059	1.497	1.932	0.155	−1.032	0.117
	(0.129)	(0.266)	(0.182)	(0.960)	(0.744)	(0.971)
RoA	0.127**	0.112*	0.102	0.147	0.127	0.115
	(0.034)	(0.064)	(0.113)	(0.282)	(0.371)	(0.426)
Leverage	0.007	0.044	0.012	−0.142	−0.061	−0.144
	(0.871)	(0.260)	(0.788)	(0.142)	(0.508)	(0.150)
BTM	−0.365	−0.492	−0.418	−2.685	−2.941	−2.729
	(0.820)	(0.759)	(0.807)	(0.463)	(0.435)	(0.474)
FCF	0.590***	0.632***	0.469*	−0.135	0.021	−0.327
	(0.008)	(0.003)	(0.055)	(0.792)	(0.967)	(0.550)
Blockholders	0.134***	0.132***	0.116**	−0.266**	−0.262**	−0.292***
	(0.004)	(0.004)	(0.020)	(0.012)	(0.016)	(0.009)
BoardInd	−0.002	0.027	0.031	0.004	0.052	0.045
	(0.943)	(0.388)	(0.358)	(0.948)	(0.484)	(0.544)
ESG-Score	−0.034	−0.046	−0.024	−0.079	−0.111	−0.063
	(0.559)	(0.419)	(0.690)	(0.545)	(0.402)	(0.648)
Intercept	37.408*	38.34*	35.107	71.679	74.923	67.996
Year Fixed Effects	Yes	Yes	Yes	Yes	Yes	Yes
N	600	600	600	600	600	600

Notes: p-values in parentheses. ***, ** and * indicates a significance level of 1 %, 5 % and 10 %, respectively.

#### 4.3.3 Additional analysis

To complement the above empirical findings, we perform several additional tests. First, we increase the requirements for the dependent variable *StratPerception*. The minimum number of analyst assessments for this additional test will be raised from 3 to 5 per year and company. Increasing the minimum feedback from analysts leads to companies with a more comprehensive assessment being considered.

Furthermore, the dependent variable *Buy* is also changed. For this purpose, the percentage of buy recommendations after three months of the annual report publication is used instead of after one month. Additionally, instead of the percentage of buy recommendations, we use the analysts’ consensus after one month and three months of the annual report publication.

The Breusch-Pagan test does not suggest that heteroskedasticity is a problem in our analysis. Nevertheless, we actively address the potential problem of heteroskedasticity by rerunning all regressions with standard errors clustered by company.

Finally, all regressions were rerun with all variables winsorized at the 98 % confidence interval. The results of all these robustness checks confirm our previously shown findings.

Moreover, our research approach is designed as a lagged analysis. First, analysts’ perceptions were obtained from mid-April to mid-June after the annual and sustainability reports for the previous financial year were published. Additionally, we retrieved the buy recommendations one month after the publication of the annual reports from Refinitv. Consequently, both independent variables, *StratPerception* and *Buy*, were obtained after the information for the other variables was available. Nevertheless, we performed lagged regression to investigate the impact of *StratPerception* and *Buy* on the integration of sustainability into corporate strategy. None of the lagged regressions shows a significant impact on sustainability integration into the corporate strategy. Therefore, there are no concerns about reverse causality.

## 5 Discussion

Overall, the results show that analysts acknowledge when sustainability is integrated into the corporate strategy (support of Hypotheses H2a) while showing an aversion to a standalone sustainability strategy (rejection of Hypotheses H1a). Thus, our results are in line with stakeholder theory, as an integrative approach to sustainability in corporate strategy has a positive impact on analysts as an important stakeholder group [36,55,99].

Our results contribute to the existing literature on the effects of a sustainability strategy that shows a positive impact on sustainability and financial performance [70,71]. Our results extend Park (2023) [40] that business strategy is important for sustainability and performance by confirming that integrating sustainability into corporate strategy is essential for analyst perceptions. The findings imply that companies with an integrated sustainability strategy meet analysts' needs [41] and provide long-term guidance by integrating sustainability into their business model to ensure future profitability [54,100].

We attribute our findings to two major problems analysts face when dealing with sustainability strategy. On the one hand, the aversion to a standalone strategy may be due to a need for more credibility and relevance of a sustainability strategy if it is separate from the corporate strategy. On the other hand, an integrated strategy might add to the corporate strategy's uniqueness and enrich the information environment, creating information costs for analysts [48], making it difficult for analysts to consider it in their company valuations.

Focusing on the credibility and relevance of a sustainability strategy, Krasodomska and Cho (2017) [86] found that Polish analysts rarely use sustainability information. They argue that sustainability information needs more quality. Accordingly, the credibility of this information is lower. The low usage could also be because analysts do not value a standalone sustainability strategy and do not attach enough importance to it compared to the corporate strategy. Furthermore, a standalone sustainability strategy might lack relevance if not integrated into the corporate strategy, as a standalone sustainability strategy might focus on different aspects of sustainability [101]. Amran et al. (2014) [102] revealed that sustainability integrated into the strategy leads to better sustainability disclosure quality. Better disclosure quality might lead to more credibility for an integrated strategy than a standalone sustainability strategy. The credibility concerns of sustainability reports [103] also might affect the credibility of a standalone sustainability strategy. To address the credibility concerns, an integrated sustainability strategy can be beneficial in terms of analyst assessments. Based on the findings of Bai et al. (2023) [104], we argue that analysts are more optimistic when they have a precise understanding of the company. Thus, analysts perceive sustainability issues better when companies actively take responsibility [104].

In addition, our results partially corroborate Rossignoli et al. (2022) [25] findings that analysts support the combination of financial and sustainability information. On the one hand, our findings show that analysts acknowledge an integrated strategy supporting the argument of a higher relevance of a sustainability strategy. On the other hand, an integrated strategy does not lead to more buy recommendations.

Another reason is the regulatory perspective. Assuming the strategy is disclosed in the management report as a component of the annual report, the auditor will audit this section. In that case, this section has higher credibility [78]. Accordingly, an integrated strategy published in the management report also has higher credibility. Conversely, the sustainability report and, thus, a standalone sustainability strategy are not audited as part of the annual report audit, leading to lower credibility with analysts and investors.

Since integrated sustainability disclosure is still voluntary, it can be argued, based on the findings of Nishitani et al. (2021) [24], that integrating sustainability into the corporate strategy is a differentiating criterion, emphasising the importance of sustainability disclosure. Therefore, our findings are consistent with the voluntary disclosure theory, as voluntary integrated sustainability disclosure improves financial analysts' accuracy [77]. Thus, we confirm a deeper insight, as sustainability strategy improves analysts’ perceptions.

The issue of information overload is widely discussed in research [105]. As sustainability grows in importance, it will be even more included in investment decision-making [14,106]. However, several barriers hinder the process of considering sustainability for investment decision-making [14]. The lack of standards for measuring sustainability performance and disclosing sustainability information by companies are the most relevant barriers to sustainability integration into investment decision-making [14,106]. Especially the latter part leads to companies not knowing which information is relevant for financial market participants, including analysts, and what has to be disclosed. Consequently, Friede (2019) [107] found sustainability information as a broadly agreed barrier in research for integrating sustainability into investment decision-making. Other issues mentioned include comparability, quantity, and quality of sustainability information [107]. These issues lead to problems in assessing the success of a standalone sustainability strategy. Accordingly, analysts show an aversion to standalone sustainability strategy as the information is not already set into the overall strategy and performance context. This argumentation supports the negative relationship between a standalone sustainability strategy and analysts’ perceptions and the issuance of buy recommendations.

Furthermore, our findings are consistent with those of Lee et al. (2018) [108], who revealed that it is difficult for analysts to make informed recommendations in a richer sustainability information environment. Analysts’ aversion to a standalone sustainability strategy may be disputed with the separation of sustainability strategy and corporate strategy. We argue that an integrated strategy is more powerful regarding whether sustainability is part of the corporate strategy. In contrast, disclosing the standalone sustainability strategy in the sustainability report is less meaningful as it is often only used as a reputation tool and contains mostly voluntary information [109].

This argument contradicts the findings of Wan-Hussin et al. (2021) [55], who found that analysts give better recommendations for companies that provide more sustainability disclosure. Their findings align with our results for a standalone sustainability strategy if a standalone sustainability strategy is not considered a better sustainability disclosure and business practice. In contrast, an integrated strategy might not impact buy recommendations because it is unlikely that the strategy would change [41]; therefore, there is no reason to consider it. Furthermore, considering an integrated strategy introduces subjectivity into analysts’ evaluations of companies, which is not desired for a company valuation [110]. Furthermore, even though analysts value an integrated strategy, they might avoid considering it in their recommendations because of its complexity and information cost in accordance with the uniqueness paradox [47,48].

To summarise, while analysts acknowledge an integrated strategy, this acknowledgement does not lead to more buy recommendations. Hsu et al. (2019) [16] showed that analysts might be optimistic about their earnings forecast for companies with higher sustainability performance. However, investors react only to adverse sustainability performance on management earnings announcements [16]. Setting the study of Hsu et al. (2019) [16] in the context of our findings suggests analysts may have been too optimistic about good sustainability performance. As a result, they have been reluctant to consider it in their buy recommendations when there is an integrated strategy, even though it is perceived positively. Another reason might be that, as previous research has shown, it takes time for analysts to develop an understanding of an integrated strategy. The better understanding then leads to a positive impact on buy recommendations [16,54].

## 6 Implications

### 6.1 Theoretical implications

Despite the limitations, our results have several implications. First, a general consideration of sustainability in research that refers to the organisational embedding of sustainability in the organisation neglects the effect of this organisational embedding. Accordingly, studies that relate to sustainability performance and reporting, for example, must always consider the organisational impact of sustainability.

Regarding research on sustainability implications for financial analysts, studies always need to have a holistic perspective. An updated information environment will face analysts with potentially easing and complexity-increasing changes. With respect to extended sustainability reporting, analysts, on the one hand, benefit from a better quantity of information but also bear the potential costs of information overload. The final synthesis of the implication on analysts will not necessarily be unitary. Depending on the context, the process of information acquisition, evaluation and weighting of specific sustainability information by analysts might differ, as shown by implications on perceptions and buy recommendations. Therefore, a holistic approach considering benefits, drawbacks and information perspectives on sustainability by analysts is required. From the lens of institutional theory, companies could be incentivised to integrate sustainability into their corporate strategy to obtain the "licence to operate" [111] and, therefore, be considered by analysts regarding sustainability issues.

### 6.2 Regulatory implications

From a regulatory point of view, there are also several implications. To reduce the issue of information overload, it seems necessary to introduce globally accepted sustainability accounting standards that companies can use to provide sustainability information in a structured manner. These standards should not promote separate reports but should be integrated into the annual report. In addition, our findings support the upcoming requirements of the European Sustainability Reporting Standards that companies need to integrate sustainability into their corporate strategies. Furthermore, to enhance the credibility of these standards, companies should be required to get external assurance for their sustainability information disclosed.

When conceptualizing sustainability reporting standards, regulatory setters should consider analysts' information acquisition and evaluation process, as analysts play a vital intermediation role for sustainability information [8]. Therefore, incorporating analysts’ perspectives in the standard concept might support a multiplicator effect on disseminating sustainability information in capital markets.

### 6.3 Managerial implications

Our results show the impact of integrating sustainability into corporate strategy. From the findings of this impact, we can support and motivate companies and managers to integrate sustainability into their corporate strategy. In general, sustainability integrated into the corporate strategy and, thus, into the entire corporate organisation is becoming increasingly important in the future. Since the transition towards a sustainable economy is one of the major missions, companies need to align business activities with environmental and social objectives. Our research indicates that companies need to integrate sustainability into their corporate strategy. Additionally, our findings aid financial market participants in understanding analysts' perceptions of a sustainability strategy and how it influences their recommendations. Only integrating sustainability into the corporate strategy positively impacts analysts. Overall, the strategic orientation, including sustainability, increases the company's value [68] and enhances access to capital [4].

Moreover, integration of sustainability into the corporate strategy could be positive for companies not only from an analysts’ perception perspective. With the increasing strategic importance of sustainability, companies need to report more strategically on sustainability in the future. Integrating sustainability into the corporate strategy might set a good baseline for future disclosure requirements. Additionally, an integrated sustainability strategy might help managers connect the dots and achieve the sustainability aspirations of the company with more strategic attention, resource allocation and governance for sustainability activities embedded in the corporate strategy.

For companies developing and implementing a sustainability strategy for the first time, the recommendation is to skip the step of a standalone sustainability strategy and immediately integrate sustainability into the corporate strategy. Companies can avoid potential negative implications on analyst perceptions and recommendations using this approach.

However, integrating sustainability into the corporate strategy alone will not necessarily lead to more buy recommendations, as our findings indicate. Companies and managers are still expected to pay attention to further pertinent corporate aspects for increasing analysts' buy recommendations. Overall, from a sustainability strategy perspective, integrating sustainability into the corporate strategy represents a good starting point to meet analysts’ perceptions.

## 7 Conclusion

### 7.1 Summary and contributions

Sustainability performance is driven by sustainability strategy [31], and analysts are increasingly considering the sustainability outcomes of companies in their assessments [8,9]. This study examines the impact of sustainability integration into the corporate strategy on analysts’ perceptions of the sustainability strategy and their buy recommendations. Using a sample of German companies listed in the DAX, MDAX and SDAX for 2017 to 2021, we provide evidence that analysts prefer an integrated strategy over a standalone sustainability strategy. Precisely, analysts show an aversion to a standalone sustainability strategy with lower levels of buy recommendations. While analysts acknowledge an integrated strategy, this acknowledgement does not lead to higher levels of buy recommendations.

We attribute our findings to the lower credibility and relevance of a standalone sustainability strategy compared to an integrated strategy. Furthermore, the sustainability information companies provide with a standalone sustainability strategy leads to an information overload, making it difficult for analysts to process all the information provided adequately. As a result, an integrated strategy is acknowledged as the companies holistically consider sustainability in their business. However, it does not lead to more buy recommendations by analysts.

This study contributes to understanding how analysts perceive sustainability information by focusing on sustainability integration into corporate strategy. Accordingly, we focus on sustainability strategy as part of the sustainability information environment. Furthermore, our study closes several research gaps. First, prior research does not focus on the impact of specific sustainability information on analysts but considers all sustainability information. Therefore, this study is the first to examine specific sustainability information – sustainability strategy – and its effect on analysts. Furthermore, we distinguish between two stages of sustainability integration into corporate strategy. We also contribute to analysts’ understanding of how they perceive these stages.

Furthermore, our results highlight the importance of sustainability management tools for integrating sustainability into strategy and management [94,106,107].

### 7.2 Limitations and future research

Our results are not free of limitation. First, analysing strategy disclosure only in the annual and sustainability reports neglects other potential sources of information such as corporate websites, investor relations websites, capital markets day presentations, and other presentations. Furthermore, we presume that companies lack an integrated sustainability strategy if they do not disclose their strategy in the annual report. However, companies may integrate sustainability into their corporate strategy without disclosing it in the annual report. Accordingly, future research should examine how the disclosure of sustainability and strategy information apart from the sustainability and annual reports affects analysts.

Because we focus on German-listed companies, our findings are transferable only to a limited degree to other countries. Therefore, future research should examine how analysts in other countries perceive different stages of sustainability integration into the corporate strategy, allowing a comparison between Germany and other countries. With the new European regulation of the Corporate Sustainability Reporting Directive (CSRD), the topic of sustainability integration into corporate strategy has gained even more significance. Therefore, further research can conduct cross-country analyses to what extent sustainability is implemented and disclosed in the strategy and business model.

Our analysis only looks at the stages of integrating sustainability into the corporate strategy without looking at the content. Accordingly, future research can be based on in-depth content analysis to investigate the impact of different strategic priorities within the sustainability strategy on sustainability or financial performance.

As our study investigates how analysts perceive different stages of sustainability integration into the corporate strategy, future research should examine how other financial market participants perceive different stages. Additionally, future research could examine what specific information analysts and investors need on sustainability.

## Data availability statement

The data associated with this study has not been deposited into a publicly available repository.

The authors do not have permission to share data.

## Additional information

No additional information is available for this paper.

## CRediT authorship contribution statement

**Toni W. Thun:** Writing – original draft, Writing – review & editing, Project administration, Methodology, Formal analysis, Conceptualization. **Anne Schneider:** Writing – original draft, Writing – review & editing, Project administration. **Christoph Kayser:** Writing – original draft, Writing – review & editing, Methodology, Formal analysis, Conceptualization. **Henning Zülch:** Software, Resources, Conceptualization.

## Declaration of competing interest

The authors declare that they have no known competing financial interests or personal relationships that could have appeared to influence the work reported in this paper.

## Footnotes

1 The questionnaire consists of several questions regarding different aspects of the company. We only used one question from the questionnaire for this study.
